# Advances in early detection of non‐small cell lung cancer: A comprehensive review

**DOI:** 10.1002/cam4.70156

**Published:** 2024-09-20

**Authors:** Nour Kenaan, George Hanna, Moustafa Sardini, Mhd Omar Iyoun, Khedr Layka, Zein Alabdin Hannouneh, Zuheir Alshehabi

**Affiliations:** ^1^ Cancer Research Center Tishreen University Lattakia Syrian Arab Republic; ^2^ Faculty of Medicine Tishreen University Lattakia Syrian Arab Republic; ^3^ Department of pathology Tishreen University hospital Lattakia Syrian Arab Republic; ^4^ Faculty of Medicine Al Andalus University for Medical Sciences Tartus Syrian Arab Republic

**Keywords:** artificial intelligence, biomarkers, EB‐OCT, LDCT, NSCLC, screening

## Abstract

**Background:**

Lung cancer has the highest mortality rate among malignancies globally. In addition, due to the growing number of smokers there is considerable concern over its growth. Early detection is an essential step towards reducing complications in this regard and helps to ensure the most effective treatment, reduce health care costs, and increase survival rates.

**Aims:**

To define the most efficient and cost‐effective method of early detection in clinical practice.

**Materials and Methods:**

We collected the Information used to write this review by searching papers through PUBMED that were published from 2021 to 2024, mainly systematic reviews, meta‐analyses and clinical‐trials. We also included other older but notable papers that we found essential and valuable for understanding.

**Results:**

EB‐OCT has a varied sensitivity and specificity—an average of 94.3% and 89.9 for each. On the other hand, detecting biomarkers via liquid biopsy carries an average sensitivity of 91.4% for RNA molecules detection, and 97% for combined methylated DNA panels. Moreover, CTCs detection did not prove to have a significant role as a screening method due to the rarity of CTCs in the bloodstream thus the need for more blood samples and for enrichment techniques.

**Discussion:**

Although low‐dose CT scan (LDCT) is the current golden standard screening procedure, it is accompanied by a highly false positive rate. In comparison to other radiological screening methods, Endobronchial optical coherence tomography (EB‐OCT) has shown a noticeable advantage with a significant degree of accuracy in distinguishing between subtypes of non‐small cell lung cancer. Moreover, numerous biomarkers, including RNA molecules, circulating tumor cells, CTCs, and methylated DNA, have been studied in the literature. Many of these biomarkers have a specific high sensitivity and specificity, making them potential candidates for future early detection approaches.

**Conclusion:**

LDCT is still the golden standard and the only recommended screening procedure for its high sensitivity and specificity and proven cost‐effectiveness. Nevertheless, the notable false positive results acquired during the LDCT examination caused a presumed concern, which drives researchers to investigate better screening procedures and approaches, particularly with the rise of the AI era or by combining two methods in a well‐studied screening program like LDCT and liquid biopsy. we suggest conducting more clinical studies on larger populations with a clear demographical target and adopting approaches for combining one of these new methods with LDCT to decrease false‐positive cases in early detection.

## 1 INTRODUCTION

Lung cancer is one of the main causes of cancer‐related fatalities globally, affecting both men and women. The World Health Organization (WHO) informs that there are 2.2 million new cases of lung cancer, which accounts for 11.7%. 1.8 million lung cancer‐related deaths were reported worldwide in 2020, which is approximately one‐fifth of all cancer deaths. This mortality rate is far greater than the combined total deaths of breast and colorectal cancers.^1^ Lung cancer can be categorized into two subtypes: small‐cell lung cancer (SCLC), which accounts for around 15% of cases, and non‐small‐cell lung cancer (NSCLC), which accounts for about 85% of cases.^2^

NSCLC can present with nonspecific symptoms. As a result, patients are frequently misdiagnosed and are thus given delayed palliative treatment. Overall, due to delayed diagnosis and treatment, the complete response and survival rates remain low in NSCLC. Patients with metastasis have a 5‐year survival rate of less than 5%.^3^

Guidelines suggest annual LDCT screening for those who are: without symptoms and age between 55 and 77, have smoked 30 packs/year or no longer, have either quit smoking in the last 15 years or are still smoking, asymptomatic individuals who age between 50 and 80, have smoked at least 20 packs/years and individuals who are asymptomatic and expected to have considerable benefit from lung cancer screening according to life expectancy estimations and verified clinical risk prediction algorithms or according to life‐year gained calculations. However, they urge against performing LDCT screening if a person has accumulated fewer than 20 pack/years of smoking, is younger than 50 or older than 80, has stopped smoking more than 15 years ago, or is not expected to have a high benefit from lung cancer screening. Moreover, LDCT screening is recommended to be avoided for people with co‐occurring conditions that significantly shorten their life expectancy and negatively affect their capability to accept treatment for early‐stage screen‐detected lung cancer or to assess the results of their screening.^4^

First‐line diagnostic procedures for NSCLC include tissue biopsy and radiographic imaging such as computed tomography (CT), positron emission tomography (PET), magnetic resonance imaging (MRI), and chest X‐ray.^5^ Chest X‐ray is incapable of identifying benign or malignant tumors when there are no rib erosions.^6^ As for MRI, The physical characteristics of the mediastinum and lungs present special difficulties.^7^ The quality and quantitative accuracy of PET images can still be impacted by a number of technological issues that arise during image capture. Particularly, one well‐known factor that can seriously affect PET imaging is the existence of respiratory motion abnormalities.^8^ Furthermore, CT scan alone had less sensitivity and specificity in staging NSCLC.^9^ However, chest low‐dose computed tomography (LDCT) has a high sensitivity, and has undergone substantial investigation. Currently, the US Preventive Services Task Force (USPSTF) recommends population‐based screening with LDCT.^10^ Furthermore, noninvasive biological biomarkers with appropriate sensitivity and specificity are required for the early detection of NSCLC.^5^ Circulating tumor DNA (ctDNA) minimal residual disease (MRD) has become a possible biomarker in recent years for predicting regression before the appearance of radiographic evidence.^2^ Despite significant improvements in early detection and treatment prospects, lung cancer patients continue to have dismal prognosis because of metastasis or recurrence.^11^ This review aims to compare several methods of early detection and explore the application of artificial intelligence (AI) and biomarkers to help identify optimal screening methods that can prevent high mortality rates in lung cancer.

## 2 METHODS

We collected the Information used to write this review by searching papers through PUBMED that were published from 2021 to 2024, mainly systematic reviews, meta‐analyses and clinical‐trials. We also included other older but notable papers that we found essential and valuable for understanding.

## 3 DISCUSSION

### 3.1 LDCT

LDCT has become a well‐recognized radiological technique for the early detection of NSCLC, given its low radiation dose. Furthermore, its cost‐effectiveness and noninvasive nature make it viable for the use of population Based Screening Programmes.^10^, ^12^ Screening for lung cancer with LDCT is recommended in high‐risk populations. High‐risk populations include people who are aged 55–74 years, have a history of smoking 30 pack/years or more, and are smoking now or have quit in the last 15 years.^13^, ^14^, ^15^, ^16^

The 2021 USPSTF proposed new recommendations on lung cancer screening from ages 55–50 years that reduced the minimum cumulative smoking exposure from 30 to 20 pack/year. Though, the yearly screening frequency, the screening age at 80 years, and the duration after smoking cessation at 15 years were left unchanged. Using a comparable modeling analysis, an economic study defined the cost‐effectiveness of the USPSTF's 2021 lung cancer screening guideline to be more cost‐effective than the 2013 USPSTF guideline when the minimum cumulative smoking exposure was reduced to 20 pack/year.^17^

The National Lung Screening Trial (NLST) included 53,439 high‐risk volunteers, which were randomly assigned to three annual screening examinations with either low‐dose CT or posteroanterior chest radiography. There was a 20% drop in lung cancer deaths following a median follow‐up of 6.5 years, with one death avoided for every 320 CT screenings. In addition to a 6.7% reduction in all‐cause mortality, the NLST is the only screening study to establish a statistically significant reduction in all‐cause mortality, owing mostly to a high proportion of fatalities (25%) caused by lung cancer.^12^, ^13^, ^14^, ^15^, ^18^ The NELSON trial randomly allocated 15,792 people to have CT screening (at baseline, 1, 3, and 5 years) or not. Lung cancer mortality was reduced by 24% after 10 years of screening, and 33%–59% after different years of follow‐up in women.^12^, ^13^, ^15^ The Multicentric Italian Lung Detection (MILD) study, another randomized controlled trial that included 4099 participants, demonstrated a reduction in the cumulative risk of lung cancer mortality by 39% over 10 years of CT screening, with one cancer‐related death evaded for every 167 screened participants.^15^

Despite LDCT's ability to reduce high mortality rates, LDCT sensitivity values can range between 43% and 100%.^10^ Moreover, many of these trials reported high false positive rates. The NLST study reported a false positive rate of 96.4%, while the Nelson study reported a rate of 59.4%. This indicates that LDCT can be nonspecific.^12^ Additionally, the false positive result will compel the patient to undergo further unnecessary medical investigations.^13^, ^14^ In order to decrease the rate of false positives, researchers developed a number of pulmonary nodule management guidelines with the goal of standardizing care. While there is some variety in the guidelines, their varied application is likely to explain the variation in false‐positive rates. When applied correctly, guidelines have been demonstrated to reduce false‐positive rates while improving sensitivity and specificity.^14^

An additional concern is high radiation exposure. However, the chest scan must be two millisieverts (mSv) or less to qualify as low‐dose.^14^ During an LDCT scan, patients receive only an average radiation dose of 1.5 mSv.^18^ Moreover, there is a compelling case for additional caution when it comes to smokers and ex‐smokers (those who have given up smoking for 4 weeks or longer), as pre‐existing lung damage puts them at a higher risk of radiation‐induced lung cancer.^14^ Nonetheless, a single‐center trial found one radiation‐induced cancer for every 108 screen‐detected lung malignancies, which amounted to a 0.05% increased risk of cancer after 10 years of screening and follow‐up imaging.^15^ According to these estimations, the increased risk of cancer after 20 yearly screening LDCT exams would be 0.12% for males and 0.22% for women, and the chance of cancer fatality would be 0.1%. These risks are minute in comparison to the projected lifetime risks of 6.7% and 5.9% for lung cancer among all U.S. smoking and nonsmoking men and women, respectively, or the estimated risk of 15% or higher for smokers.^15^

Another issue with LDCT scans is overdiagnosis, which is a term used to describe cancer found by screening that an individual would not have had symptoms or damage throughout their lifetime if they had not undergone the screening process.^14^ Analysis of the original NLST data revealed that adenocarcinomas, which were categorized as bronchioloalveolar cell type at the time, accounted for 18% of the screen‐detected malignancies in the LDCT cohort, suggesting that they may have been overdiagnosed. Furthermore, studies using statistical modeling to simulate lengthier follow‐up predicted that 10% of cases will be overdiagnosed.^15^ A similar pattern was noted in the NELSON trial, which revealed an overdiagnosis rate of 19.7% at 10 years, dropping to 8.9% during 11 years of follow‐up.^14^ It should be emphasized that after a cancer has been identified and treated as a consequence of screening, it is impossible to tell if the disease would have manifested clinically in the absence of screening. Consequently, it is difficult to say if a specific cancer was overdiagnosed.^15^

One notable prospective cohort study is the TALENT study (Taiwan Lung Cancer Screening for Never Smoker Trial) done at 17 tertiary medical centres and sponsored by The Ministry of Health and Welfare, Taiwan. Eligible individuals were aged between 55 and 75 years, never‐smoker or had smoked fewer than 10 pack‐years and stopped smoking for more than 15 years (self‐report), and having one of the following risks: family history of lung cancer within third‐degree, passive smoking exposure, TB/COPD, cooking index ≥110, or cooking without using ventilation. From Feb. 2015 to July 2019 a total of 12,011 individuals were enrolled and went through LDCT screening according to the guideline suggested by ACR. Among all participants 2094 (17.4%) were positive, 318 (2.6%) of them were diagnosed with lung cancer (257 [2.1%] participants had invasive lung cancer and 61 [0.5%] had adenocarcinomas in situ). The prevalence of invasive lung cancer was higher among participants with a family history of lung cancer (161 [2.7%] of 6009 participants) than in those without (96 [1.6%] of 6002 participants). Furthermore, a positive LDCT scan had 92.1% sensitivity, 84.6% specificity, a PPV of 14.0%, and a NPV of 99.7% for lung cancer diagnosis. This is an interesting study that detected highly invasive lung cancer at 1 year after the baseline LDCT scan. However, interpretations suggest that overdiagnosis could have occurred, particularly in participants diagnosed with adenocarcinoma in situ, and further research on risk factors for lung cancer is required, especially for individuals without a family history of lung cancer.^19^, ^20^

### 3.2 Endobronchial‐assisted diagnosis

Recently, bronchoscopy has been widely used to detect lung cancer and guide biopsy aspiration. However, bronchoscopy encounters diagnostic constraints, especially in pre‐malignant lesions measuring between 0.2 to 1 mm in thickness and possessing a diameter of a few millimeters.^16^ Hence, several devices have been developed that combine precise optical imaging techniques with real‐time vision provided by bronchoscopy. Endobronchial optical coherence tomography (EB‐OCT) has been well known for early detection and diagnosis of pulmonary fibrosis and other interstitial lung disease.^21^ This precise imaging method accompanies both real‐time bronchoscopic vision and three‐dimensional imaging of OCT using high resolution of 10–20 μm waves. By using low‐coherence interferometry, it is possible to obtain High‐resolution horizontal and vertical images of airways through analysis of the reflecting light.^21^, ^22^, ^23^

**FIGURE 1:**
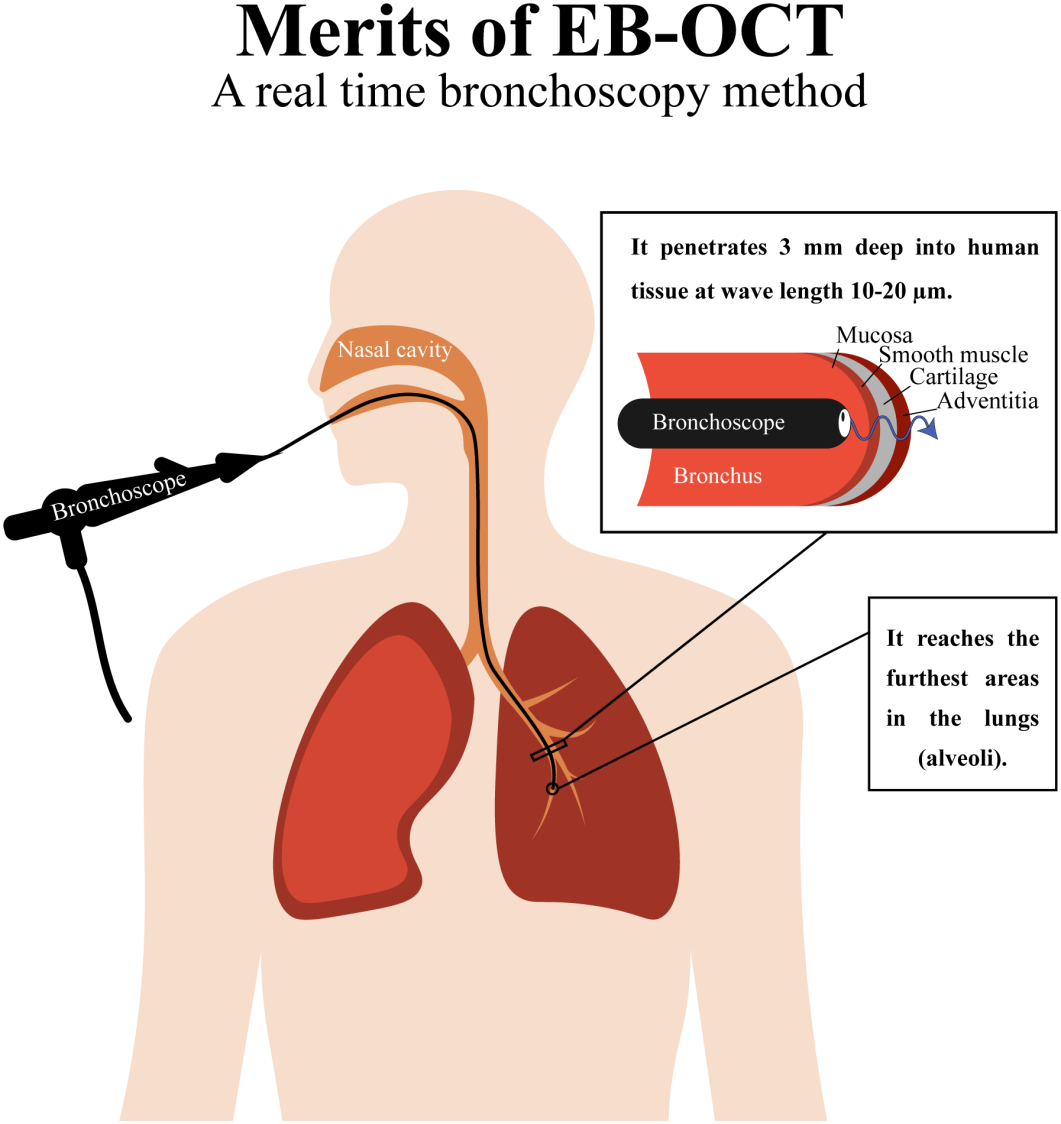
Due to its high resolution of 10–20 μm waves Endobronchial optical coherence tomography (EB‐OCT) can generate high‐resolution longitudinal and cross‐sectional images of airways and display and measure the mucosal layer, submucosal layer, adventitia, alveoli, glands, and cartilage of the bronchial wall with a penetration depth of 3 mm.

Using EB‐OCT in the early detection of lung cancer proved to have several merits. The near‐infrared light band is harmless to patients. EB‐OCT probes can also display and measure the mucosal layer, submucosal layer, adventitia, alveoli, glands, and cartilage of the bronchial wall. Moreover, EB‐OCT examination can be conducted in conscious patients. Therefore, anesthesia and mechanical ventilation risks are alleviated.^22^ This technique is also an excellent method for accessing small and distant bronchioles. Because of their flexibility and minuscule measurement, the probes can enter large, medium, and small airways up to 1–9 bronchi (0.9 mm), which facilitates diagnosing early pathological changes associated with various lung diseases and peripheral masses such as adenocarcinoma^22^, ^23^ (Figure 1).

During CT screening, a considerable number of patients could have false‐positive findings. Moreover, even if CT findings are determined as pathological, different diagnoses including pneumonia and lung cancer subtypes (adenocarcinoma, squamous cell carcinoma [SCC] and small cells lung cancer) can have similar appearances on CT imaging. To overcome difficulties pertaining to CT imaging, a clinical trial, which included 31 patients with pathological pulmonary nodules, found that integrating EB‐OCT along with machine learning algorithm increased sensitivity, specificity, and accuracy of diagnosing malignant pulmonary nodules to 90.41%, 77.87%, and 83.51%, respectively.^24^ Another study that performed EB‐OCT on 111 resected specimens of solitary pulmonary nodules (usually require transbronchial needle aspiration to detect malignancy) followed by imaging were evaluated in blinded assessments has demonstrated a sensitivity and specificity of 95.4% and 98.2%, respectively.^25^ One clinical trial included 138 smoking volunteers and 10 patients with lung cancer. A total of 281 OCT images and the corresponding bronchial biopsies were acquired. The histopathology of 145 normal/hyperplasia, 61 metaplasia, 39 mild dysplasia, 10 moderate dysplasia, 6 severe dysplasia, 7 carcinoma in situ, and 13 invasive carcinomas were demonstrated.^26^ Ultimately, one prospective clinical trial published in 2022 that included 135 patients who underwent EB‐OCT between February and December of 2019 in Chinese PLA general hospital showed a 94.1% accuracy, a 97.1% sensitivity, and a 83.3% specificity in distinguishing between benign and malignant bronchial lesions. The inclusion criteria were: volunteering, age ≥ 18 years old, current routine clinical diagnostic bronchoscopy, normal ECG, and sufficient hematopoietic function. Additionally, it demonstrated 95.6% accuracy, 96.2% sensitivity and 93.8% specificity in diagnosing (SCC). An accuracy, sensitivity, and specificity of 94.3%, 89.5%, and 100%, respectively, was observed in differentiating adenocarcinomas.^27^ These studies raise hopes for EB‐OCT as a promising method for improving the accuracy of early detection of lung cancer.

EB‐OCT is still not an adopted screening method, which is predominantly due to multiple limitations. For instance, most EB‐OCT images of adenocarcinoma, SCC, and poorly differentiated cancer were conducted in vitro. Moreover, it is well known that tissue degeneration and changes in blood flow could largely affect the accuracy of OCT images. Furthermore, the small sample size in these studies diminishes the capacity of using this procedure in a population‐based screening program. Finally, most EB‐OCT image features lacked consolidated evaluation standards.^27^ Thus, there is a clear need for larger clinical trials to prove the efficiency of using EB‐OCT and to determine a standard criterion for evaluating pathological images.

Bronchoscopy has been combined with other techniques, one of which is confocal laser endomicroscopy. A notable original research was published in 2022 to study the feasibility and safety of Needle‐based confocal laser endomicroscopy (nCLE) for real‐time differentiation between malignancy and airway/lung parenchyma. The study performed nCLE on 26 patients with suspected peripheral lung cancer. Researchers found that in 24 patients (92%) good to high‐quality videos were acquired (detected malignancy in 22 out of 23 patients with lung cancer) with a 95% accuracy. That could indicate that nCLE imaging of peripheral lung lesions is practicable, safe and allows real‐time lung cancer detection.^28^

Confocal laser endomicroscopy was also introduced as a diagnostic method for visceral pleural invasion (VPI) which is an important prognostic factor in lung cancer. A study included 35 patients with primary lung cancer who underwent surgery between April 2018 and August 2019. Researchers recorded the area where pleural change was noted via a short video using CLE. Another validation study for intraoperative VPI according to the cutoff value of the defect ratio of the autofluorescence‐positive structure was done. For the defect ratio of the autofluorescence‐positive structure, the areas under the receiver operating characteristic curve were 0.86–0.91. As a predictor of VPI, the operating defect ratio of autofluorescence‐positive structure cutoff of ≥50%, with sensitivity of 83.3%–100.0%, specificity of 57.7%–73.1%, positive predictive value of 35.3%–41.7%, negative predictive value of 95.0%–100.0%, and accuracy of 75.0%–78.1%. Finally, in the validation study, the sensitivity, specificity and diagnostic accuracy rate were 100%, 83.3%, and 86.7% respectively.^29^

Another paper was published in 2023 to study nCLE for real‐time microscopic imaging during robotic bronchoscopy in small peripheral lung nodules. Twenty patients were included and underwent fluoroscopy and radial EBUS‐assisted robotic bronchoscopy. After that, nCLE‐imaging of the target area and a biopsy needle were performed. The results showed good‐quality nCLE‐videos. In nine patients (45%) inadequate positioning of the needle and repositioning was performed. After that, nCLE‐imaging provided tool‐in‐nodule‐confirmation in 19/20 patients. Subsequent ROSE revealed representative material in 9/20 patients (45%) and the overall diagnostic outcome was 80% (16/20). Two cases were diagnosed with malignancy during follow‐up from the three patients with malignant nCLE‐imaging but insufficient pathology.^30^

Furthermore, rapid assessment for lung core needle biopsies could be highly required especially during the operation room. Hence, many techniques may help with this, including polarization sensitive optical coherence tomography (PS‐OCT). One study published in 2021 was conducted at Massachusetts General Hospital, where researchers obtained 42 fresh, intact CNB specimens (ranged from 2 mm to 8 mm in length and 0.4 mm to 1 mm in thickness) from excised surgical lung nodule resection specimens and immediately imaged with benchtop PS‐OCT in a thin layer of phosphate buffered saline solution to prevent tissue drying. Histological results showed that 33 CNB possessed some amount of tumor (mean: 10.1%, SD: 15.2%, range: 0.3%–70.6%), 42 CNB possessed fibrosis (mean: 22.4%, SD: 14.4%, range: 4.3%–63.6%), and 16 CNB possessed normal lung parenchyma (mean: 15.3%, SD: 28.7%, range: 4.8%–90.1%). There were no observable tumor cells present in 9 CNB specimens, which consisted totally of fibrosis and/or normal lung parenchyma. However, PS‐OCT could have several limitations as mentioned by researchers, including overestimating or underestimating the amount of tumor. They also suggest conducting future studies using 3D histology to perform a more in‐depth analysis comparing volumetric PS‐OCT data against 3D histology.^31^

One significant publication that aimed to study combining endobronchial ultrasound‐guided fine‐needle aspiration (EBUS‐FNA) with rapid EGFR/KRAS analysis. Three hundred and ninety‐eight patients with lung cancer were included in this study from May 2011 to December 2017 and underwent EBUS‐FNAs with rapid on‐site evaluation. Then researchers studied mutations with Sanger or new generation sequencing. Another 43 cases were tested with a fully automated real‐time PCR rapid technique. ALK abnormalities were observed by immunohistochemistry and/or in situ hybridization. The genotypic result could be acquired in 316 cases (79.4%) and in 180 of the 198 more recent cases (90.9%). Genetic mutations were observed in 191 cases (48.0%). Researchers obtained EGFR/KRAS mutations within a few hours after the histological diagnosis of the EBUS‐FNA by analyzing fresh specimens after intra‐operative cytological diagnosis. These findings indicate that using EBUS‐FNA can provide high‐quality biological material comparable to other clinical sampling methods.^32^

### 3.3 Liquid biopsy and biomarkers

For a long time, clinicians have relied on tissue biopsy to diagnose lung cancer. However, this invasive and traumatic procedure involves many risks. As a result, using liquid biopsies is gaining popularity in clinical practice because of its noninvasiveness and easily attainable quality. In addition, a liquid biopsy involves acquiring a small amount of blood to reflect the full state of the tumor, which provides real‐time monitoring and indicating changes earlier than normal detection methods. This technique safely promotes accuracy and specificity in early detection.^33^, ^34^ Furthermore, liquid biopsies allow for the detection of many biological and immunological markers.^33^, ^35^ We have summarized the most prominent biomarkers involved in the detection of NSCLC below.

#### 3.3.1 RNA molecules detection (miRNA—circRNA)

MicroRNA (miRNA) plays an essential role in regulating various gene expressions including those which lead to cells proliferation, differentiation, and apoptosis. It is well known that malignancy is often accompanied by the occurrence of several mutations. Thus, detecting certain miRNA molecules and mutations could be a promising method for NSCLC early diagnosis. In fact, miRNAs are very stable in body fluids, including serum and plasma. Detection of miRNA can be highly sensitive. Nevertheless, the expression of miRNA can exhibit tissue‐specific behavior.^12^ miRNAs can work as cancer suppressors^12^, ^35^ affecting protective genes, such as PTEN and TP53. When dysregulated, miRNAs can also increase in the expression of oncogenes like RAS and MYC, leading to increased tumorigenesis.^12^ Kirsten rat sarcoma viral oncogene homolog (K‐Ras), tumor protein p53 (TP53), MET, B‐Raf proto‐oncogene, serine/threonine kinase (BRAF), ERBB2, the anaplastic lymphoma kinase (ALK), ROS proto‐oncogene 1 receptor tyrosine kinase (ROS), RET, and NTRK rearrangement are all driver gene alterations that have been detected in NSCLC patients.^35^

Several trials suggested that combining two or more gene methylation methods have increased sensitivity and specificity. One pragmatic study published in 2023 aimed to evaluate detecting RNA extended‐panel in samples taken via endobronchial ultrasound‐guided fine needle aspiration. The study included a total of 292 patients and 300 cases of nonsquamous NSCLC with mediastinal metastasis were diagnosed with EBUS procedures from 2016 to 2019 at the University of Pennsylvania. Results showed a success rate of 91.5% in samples with >10% tumor cellularity. Conversely, 92.8% of RNA next generation sequencing (NGS) panels reported no fusion genes.^36^ Another case–control study which included 140 NSCLC patients and 140 healthy controls used a suggested panel integrating CTC level in blood and 5 mRNA biomarkers in saliva (CCNI, epidermal growth factor receptor [EGFR], FGF19, FRS2, and GREB1). The study found the method capable of differentiating NSCLC patients with a sensitivity and specificity of 92.1% and 92.9% in the discovery phase, and a sensitivity and specificity of 88.3% and 90.0% in the validation phase, respectively.^37^ In addition, a previous study has proposed that using two‐phase screening, one highly sensitive miRNA panel followed by highly specific one to detect stage I and II NSCLC could increase the overall detection sensitivity to 91.6% with an overall specificity of 93.4%. This study validated this method on a set of 204 pathologic samples and 247 cytologic samples.^38^

In comparison, circRNAs are considered novel specific diagnostic markers for NSCLC, which carry significant specificity and sensitivity.^39^ More than 1000 circRNAs are present in human serum exosomes. One molecule which is a circular RNA mediator of cell motility (circ‐MEMO1) has been identified as an oncogene in NSCLC,^40^ and has been shown to be increased in exosomes generated from the serum of NSCLC patients. The AUC reached a value of approximately 0.76, with a diagnostic sensitivity and specificity of 56.67 and 96%, respectively.^39^ In a recent paper published in 2022, formalin‐fixed paraffin‐embedded (FFPE) lung cancer tissues were retrospectively collected, including 27 early‐stage and 26 late‐stage cancer patients. A custom‐made panel of 78 circRNAs was produced, which also included six linear reference genes (GAPDH, MRPL19, PSMC4, RPLP0, SF3, and UBB) and four mRNAs of FAM13B, HIPK3, MGA, and UBXN7 genes. The results provided a positive‐predictive value (PPV) and negative‐predictive value (NPV) of 92.6% and 87.5%, respectively, whereas accuracy, sensitivity, and specificity were of 90.6%, 92.6%, and 87.5%, respectively, using RFE algorithm.^41^

From another perspective, immunohistochemistry (IHC) plays an increasing role in detecting lung cancers and separating cancer subtypes due to its efficiency in analyzing subsequent molecules.^42^ Based on the latest studies, recommendations for the best practical usage of IHC in lung cancer diagnosis considered Thyroid Transcription Factor 1 (TTF‐1) when it is detected by 8G7G3/1 monoclonal antibody and p40 as the best biomarkers for NSCLC subtypes but are not considered as diagnostic. Napsin on the other hand is considered as the second best marker for adenocarcinoma.^43^ One recent study consisted of 268 pre‐surgical resections for primary lung carcinomas in Lund and 270 in Stockholm were designed to compare IHC stains using the Mann–Whitney U test and the Kruskal–Wallis test through multiple regression analysis on a retrospective follow‐up of a 15‐month period. This study has found that 164 (61%) of 268 and 174 (64%) of 270 cases in the Lund and Stockholm cohorts had a pre‐surgical diagnosis, respectively. The results revealed that diagnostic inaccuracy was often associated with a squamous marker not included in the IHC panel, including TTF‐1 expression in SCCs (with clone SPT24). Hence, application of both TTF‐1 (preferably clone 8G7G3/1) and p40 would carry an accurate specified diagnosis thus they are recommended in low morphology NSCLC.^44^

These previous studies prove the promising future of RNA molecules and genetic molecules in discrimination of NSCLC subtypes as an early method. However, most of these studies encounter numerous limitations. For instance, RNAs molecular diagnostic accuracy for nonmalignant cases and metastasis to the lung were not evaluated. Thus, more future trials are needed to assess the effectiveness of these molecules, and combine multiple markers to raise sensitivity and specificity.^12^, ^44^

#### 3.3.2 Circulating tumor cells (CTCs)

CTCs were introduced in 1869 as tumor cells that break off primary or metastatic lesions and enter peripheral circulation spontaneously or as a result of diagnostic/treatment.^33^ It may be possible to utilize CTCs as a minimally invasive biomarker to characterize the tumor by detecting associated somatic mutations and copy number aberrations. Additionally, CTCs may be a helpful tool in the identification of metastatic cancer because of their ability to enter venous or lymphatic veins and seed in many organs.^12^

Hofman and colleagues found that CTC measurement for lung cancer early detection was promising in early and experimental trials. The same research group then launched a large multicenter prospective trial (NCT02500693), which enrolled a cohort of 614 high‐risk subjects according to the NLST criteria, to assess the diagnostic accuracy of CTCs detected by the isolation by size of epithelial/tumor cells (ISET) technology. CTC analysis had a poor sensitivity of around 26% in detecting 19 lung tumors which were detected on the first (LDCT) scan.^45^ Another recent study done on patients with (*n* = 107) and without lung cancer (*n* = 100) was conducted using the 4‐color FISH test on the peripheral blood mononuclear cell (PBMC) fraction recovered by density gradient centrifugation. This approach yielded encouraging detection rate findings and identified cells with at least two polysomies or gains at 4 loci relevant in NSCLC cancer development or prognosis (i.e., at 10q22.3, 3p22.1, 3q29 loci, or chromosome 10 centromere) in 89% of 107 patients with ≤30 mm diameter pulmonary nodules. In contrast, none of the 100 lung cancer free control cases tested positive when the cut‐off value was ≥3 cells with genomic aberrations. The majority of lung cancer patients were early stage (67% were stage I or II) and the primary histological subtype of NSCLC was adenocarcinoma. In addition, the overall sensitivity, specificity, and accuracy were 89%, 100%, and 94.2%.^46^ One meta‐analysis on 27 studies (reporting 2957 patients) demonstrated that CTC values were affected according to the histological subtype of cancer. SCLC patients (overall survival HR 3.11, 95% CI 2.59–3.73) had worse prognostic values compared to NSCLC patients. Moreover, the rarity of CTCs compared to the multitude of nonmalignant blood cells poses the need for enrichment techniques, which are limited by the heterogeneity of CTCs. These low levels of CTCs found in patient blood are mainly responsible for detection problems and imply the need for repeating the blood drawn up to three consecutive times in one‐ or two‐day intervals to detect the escaping tumor cells from the immune system. In other words, the larger the blood volume analyzed, the greater the chances are for detecting CTCs in a larger number of samples.^47^

Some trials have suggested that it may be possible to combine CTCs and LDCT to achieve an early diagnosis of NSCLC. A trial has been done on a total of 80 people that helped diagnose 29 NSCLC patients, 31 high‐risk lung cancer screening subjects on LDCT, and 20 healthy never‐smokers. Nonclustered CTCs were detected in all NSCLC patients, whereas CTC clusters were discovered in 12 out of 29 (41.4%) NSCLC patients. Importantly, none of the 31 high‐risk screening participants included CTC clusters. However, nonclustered CTCs were identified in 18 out of 31 screening individuals (58.1%). There were no CTCs found in the 20 healthy, never‐smoking control participants. These findings indicate that CTC clusters are limited to a subgroup of NSCLC patients and are not seen in high‐risk screening participants with or without a benign lung nodule on LDCT. Therefore, because of their specificity to NSCLC, the CTC clusters have potential importance as liquid biomarkers in LDCT screening.^48^

#### 3.3.3 Circulating DNA methylation (ctDNA)

Circulating tumor DNA (ctDNA) has garnered increasing attention as a noninvasive cancer diagnostic method. Tumor cells release ctDNA into the bloodstream and other biofluids, which can be found and measured using molecular methods.^49^, ^50^ Tumor cells may release ctDNA by a variety of processes, including apoptosis, necrosis, and active vesicle transport.^49^, ^50^

Between 0.01% and 90% of the total circulating cell‐free DNA (ccfDNA) in cancer patients' blood may be made up of ctDNA. However, ccfDNA is a quick, dependable, economical, and least intrusive method. Thus, it is possible to track the development of cancer in real‐time and more accurately reflect the diverse genetic makeup of each tumor subclone. Additionally, research has demonstrated that these molecules include tumor's mutations and epigenetic changes. As an epigenetic‐based diagnostic method, aberrant DNA methylation has garnered attention due to its early onset, cancer specificity, biological stability, and accessibility in physiological fluids. These factors make it a desirable target to investigate in liquid biopsies.^49^

Methylated ctDNA has demonstrated to be a potential biomarker for cancer diagnosis and prognosis. Methylated ctDNA is a circulating DNA with methyl groups added to CpGs.^49^ In order to create 5‐methylcytosine (5mC), this epigenetic process involves the covalent attachment of a methyl group donated by S‐adenosylmethionine (SAM) to the 5‐position carbon of a cytosine ring. DNA methyltransferase enzymes (DNMTs), specifically DNMT3a, DNMT3b, which catalyze de novo DNA methylation during embryonic development and establish tissue‐specific DNA methylation, and DNMT1, which is frequently linked to the maintenance of methylation patterns during replication, are responsible for this modification.^49^ This mechanism usually affects cytosine residues found at CpG dinucleotides, which are frequently found in big clusters called CpG islands. These islands are mostly found at the 5′ end of genes and account for around 60% of the promoter regions of human genes.^49^ Methylated ctDNA has varying sensitivity and specificity across investigations. One meta‐analysis included 33 studies which considered methylated ctDNA and 40 different genes, including the most frequent SHOX2, RASSF1A, and APC. The sensitivity ranged from 8% to 93%, while the specificity ranged from 69% to 100%.^49^ Another systematic study registered by York University included 25 case–control studies and assessed their quality using the Quality Assessment of Diagnostic Accuracy Studies 2 (QUADAS‐2) tool. The diagnostic sensitivity and specificity of methylated ctDNA for lung cancer varied significantly depending on the included gene's number. Indeed, the sensitivity ranged between 0% and 97% using a four‐gene panel. On the other hand, specificity ranged from 8% to 100%. All methods that combine several panels found an increase in diagnostic sensitivity. For instance, Wen 2021 validation HOX49 demonstrated an 80% sensitivity and 53% specificity. Additionally, Roncarati 2020 used a combination model that had around a 97% sensitivity and a 74% specificity.^51^

Respiratory secretions, such as sputum, bronchoalveolar washings, and pleural fluid, are more sensitive in the diagnosis of lung cancer compared to plasma. Evaluating sputum samples has the potential to enhance existing screening algorithms and increase the sensitivity of methylation‐based methods for detecting lung cancer. The discovery that promoter hypermethylation in sputum samples predicted the development of lung cancer in higher‐risk individuals showed the promise of sputum‐based epigenetic testing. Out of the 14 genes whose promoter methylation was investigated, six were linked to a risk of lung cancer that was >50% higher. Furthermore, in sputum samples taken within 18 months of a cancer diagnosis, concurrent methylation of three or more of the six genes was linked to a 6.5‐fold higher risk of lung cancer development (95% CI: 1.2–35.5%), That is a 64% sensitivity and 64% specificity.^50^

Breathomics is a rapid, sensitive, specific, and minimally invasive method for studying volatile organic compounds and inorganic gases endogenously produced and released as a result of metabolic pathways associated with body functions.^52^ In this regard, studies investigated Exhaled Breath Condensate (EBC) as a noninvasive way to sample the quantitation of miRNAs secreted by lung cells. One study conducted EBC samples from 20 treatment‐naïve patients with pathologically confirmed lung cancer and profiled 20 healthy subjects for miRNAs expression. After validating selected miRNAs using quantitative‐PCR, in an independent set of 10 subjects from both groups a total of 78 miRNAs were found to be remarkably upregulated in the EBC of lung cancer patients compared to the control group. From 78 miRNAs six of them were shortlisted for validation. Of these, miR‐31‐3p, let7i, and miR‐449c were significantly upregulated and exhibited good discriminatory power.^53^

Although research on methylation biomarkers for cancer detection has grown exponentially. One significant drawback of using methylation‐specific PCR‐based methods for early cancer diagnosis is that individuals with early‐stage malignancies have lower plasma levels of ctDNA.^50^ As a result, highly specific and sensitive detection techniques are required. In addition, the smaller the number of test samples, the higher the specificity and sensitivity of a small number of markers may not be accurate. Therefore, before any marker is used clinically, large‐scale sample studies ought to be carried out.^54^ According to reports, smaller tumors have less central necrosis, less vascularity, and less lymphatic invasion, which lowers the amount of ctDNA fragments in blood.^50^ In order to validate the clinical use of epigenetic biomarkers, larger multicenter studies are necessary regarding the applicability in routine clinical practice and post‐operative monitoring.^49^, ^50^

Circulating DNA fragmentomics is a novel method for cancer screening that has been receiving increasing interest recently. High fragmentation of nuclear circulating DNA (cirDNA) is defined as chromatin being organized and protected by mononucleosomes. Fragmentomics considers size pattern characterization, the positioning and occupancy of nucleosomes and can determine the tissue of origin hence distinguishing cancer‐derived cirDNA. Precise parametrics allow us to highly distinguish between cancer patients and healthy individuals mainly based on single fragment length, frequency of unique reads at single‐base resolution, the difference of frequency of two single fragment lengths at 1 bp resolution, the size range fraction frequency, or the difference of size range ratio.^55^

A recent research conducted PacBio SMRT sequencing data of plasma cfDNA, including healthy individuals (*N* = 15), pregnancies of different trimesters (*N* = 28), HBV carriers (*N* = 13), and patients with HCC (*N* = 13) from previous studies, collected blood samples from healthy individuals (*N* = 5), HBV carriers (*N* = 6), and patients with HCC (*N* = 35) from the Prince of Wales Hospital, Hong Kong, with written informed consent. Additionally, mice with a CRISPR‐Cas9‐targeted deletion of exon 5 in Dnase1l3 of the C57BL/6N background were generated by The Jackson Laboratory. Researchers proposed a plasma DNA fragmentation model based on the end motifs of cfDNA molecules to investigate the origins of long cfDNA molecules from different genomic elements. Results found a stronger association between the abundance of long molecules and mRNA gene expression levels, compared with short molecules (Pearson's *r* = 0.71 vs. −0.14). In addition, surrounding CpG sites long and short molecules show distinct fragmentation patterns. The results from mice samples showed that the proportions of long molecules originating from transcription start sites are lower in Dffb‐deficient mice but higher in Dnase1l3‐deficient mice compared with that of wild‐type mice.^56^

Another study conducted cfDNA fragmentomic features from 191 whole‐genome sequencing data and studied them in 396 low‐pass 5hmC sequencing data that contained four common cancer types and control samples to prove the diagnostic potential of combining epigenetic markers and fragmentomic information of cell‐free DNA for detecting various types of cancers. Results showed aberrant ultra‐long fragments (220–500 bp) that countered normal samples in size and coverage profile. These fragments leveraged the ability to detect cfDNA hydroxymethylation and fragmentomic markers simultaneously in low‐pass 5hmC sequencing data with sensitivity and specificity of 88.52% and 82.35%, respectively.^57^

However, cirDNA fragmentomics encounters numerous hurdles. For instance, potential contamination of the cirDNA extract by blood cell genomic DNA, the increasing cost and time needed to produce the analytical data using the NGS‐based methodology, the implementation of multimodal analysis from cirDNA relies on standard high‐throughput sequencing, which requires specific protocols for the investigation of different classes of biomarkers. Nevertheless, mapping and sequencing bias, inadequate read depth, GC content, PCR amplification, the choice of reference genome, and k‐mer composition can all make the identification of fragmentome characteristics more challenging. Moreover, we still lack knowledge on biological confounders which limits the implementation of most technological approaches. Further, this method is currently limited in early‐stage cancer screening hence, the sensitivity of most cirDNA‐based methods in detecting stage I or II cancer has been low (<30%). Not to mention that strategies which rely on machine learning classification, which is often used to analyze the data, can be biased by the quality of the control cohort. In other words, cirDNA fragmentomics is a promising method that is worth mentioning but still, it counters various limitations that require more studies.^55^

#### 3.3.4 Metabolic change as an early diagnostic method

Cancer cells require adaptations to thrive and progress. Consequently, they undergo metabolic transformations to satisfy their energy and synthesis demands for survival.^58^ Oxygenated tumors exhibit unique metabolic characteristics in which they tend to ferment most of their glucose consumption into lactate rather than oxidizing pyruvate in their mitochondria. This phenomenon is known as the “Warburg effect,” which is a suggested to be the result of mitochondrial activity saturation.^59^ However, even cancers that rely on lactate production instead of aerobic oxidation can revert to aerobic metabolism and use tricarboxylic acid (TCA) cycle metabolites for synthesis and anabolism.^60^

Alterations in metabolic pathways can result in significant changes in the concentration of biological metabolites within tissues and biofluids such as plasma, urine, and bronchial aspirate.^61^ A new fluid, bronchoalveolar lavage fluid (BALF), has been recently introduced for detecting lung cancer.^62^ In the context of lung cancer, more than 150 metabolites have been identified in the various altered metabolic pathways of cancer cells. The most‐studied metabolites were: glucose, lactate, pyruvate, glutamine, glutamate, leucine, isoleucine, valine, citrate, acetate, fumarate, tyrosine, histidine, ornithine, arginine, creatinine, choline, VLDL, fatty acids, glycerol, ketone bodies.^61^

A systematic review and meta‐analysis showed that three amino acids (methionine, tryptophan, and proline), smoking‐related metabolites, folate, sialic acid, and creatine riboside had a statistically important relationship with lung cancer risk.^63^ Zhu et al. defined lipid metabolism‐related genes (LMRGs) that are associated with recurrence and long‐term survival of early‐stage lung adenocarcinoma, creating the first lipid metabolism‐based signature which can predict recurrence.^64^

One recently published prospective observational study conducted between Sept 1, 2020 and Dec 31, 2020 in Peking University People's Hospital in China selected 28 volatile organic compounds (VOCs) as candidates based on a literature review and used High‐pressure photon ionization time‐of‐flight mass spectrometry for breathomics testing before surgery and 4 weeks after surgery and then performed multivariable logistic regression to establish diagnostic models based on selected VOCs. Finally, an external validation was conducted to evaluate the performance of these VOCs for lung cancer diagnosis. Results showed that from 84 patients with lung cancer, perioperative breathomics demonstrated 16 VOCs as lung cancer breath biomarkers (Aldehydes, Hydrocarbons, Ketones, Carboxylic Acids, And Furan). In the external validation study that included 157 patients with lung cancer and 368 healthy individuals, after adjusting for age, sex, smoking, and comorbidities, patients with lung cancer showed elevated spectrum peak intensity of the 16 VOCs. Lastly, the diagnostic model including 16 VOCs achieved an area under the curve (AUC) of 0.952, with sensitivity, specificity and accuracy of 89.2%, 89.1%, and 89.1% respectively. While diagnostic model including the top eight VOCs showed an AUC of 0.931, sensitivity of 86.0%, specificity of 87.2%, and accuracy of 86.9%.^65^

ZEUS (ZIF‐based electrochemical ultrasensitive screening) is a new device for isopentane analytics with focus on lung cancer diagnosis based on the measurement of volatile organic compounds (VOCs) and gases that are produced by the body because of the metabolic pathways. The developed device shows sensitivity and specificity for the detection of isopentane up to 600 parts‐per‐billion. Researchers purchased analytical grades of chemicals such as zinc nitrate, and 2‐methylimidazole required for ZIF‐8 synthesis from Sigma‐Aldrich and used them without further purification and performed FESEM, FTIR, and DLS for both [BMIM]BF4@ZIF‐8 and ZIF‐8 (control compound) to analyze the physico‐chemical properties of the synthesized nanocomposite. The results mainly demonstrate sensing capability up to 600 ppb as the level of detection with high specificity and sensitivity in sensor performance.^52^

Metabolic change detection is a promising method for early detection of lung cancer due to its noninvasive nature and lack of radiation exposure. However, these studies exhibit significant variability. Further studies need to identify specific metabolites that can be applied in clinical practice.^61^, ^62^

### 3.4 Artificial intelligence‐assisted diagnosis

AI began to impact clinical practice, especially in the field of medical imaging. Manual detection methods may miss small nodules (<10 mm in diameter). Therefore, computer‐aided diagnosis systems that use convolutional neural network (CNN)‐based deep learning (DL) approaches are important for quicker and more reliable diagnoses.^66^ The introduction of AI‐powered imaging models has enabled automated lesion detection, characterization, segmentation, and risk prediction for diagnosis and outcome assessment.^67^ With the latest developments of deep learning, a variety of computer‐aided diagnostic tools were allowed by data from the National Lung Cancer Screening Trial (NLST).^68^

**FIGURE 2:**
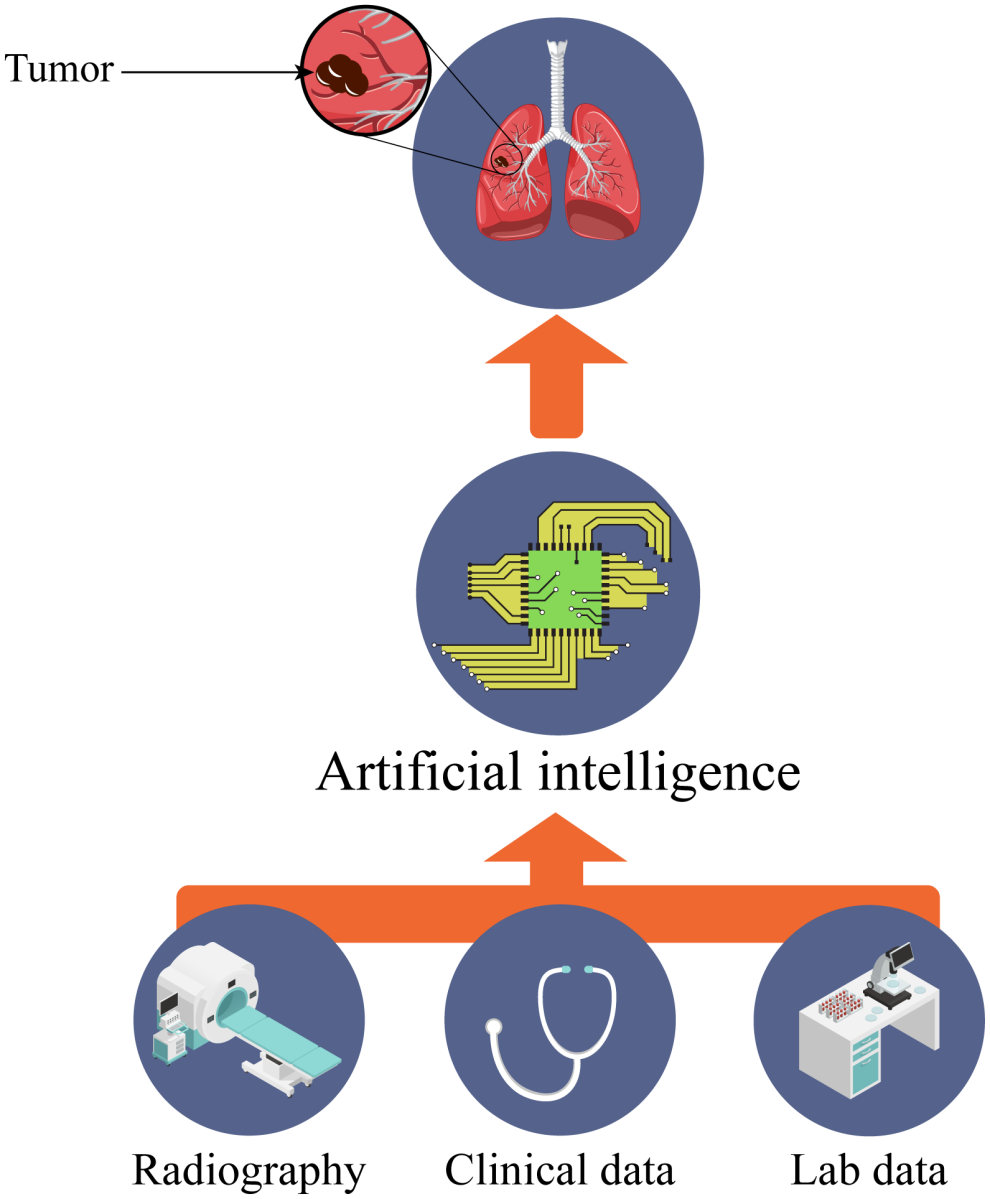
The integration of artificial intelligence (AI) with medical engineering, diagnostic and screening techniques has provided significant advancements in the medical field. AI can enhance CT scans, tomosynthesis and breath tests. Moreover, digital pathology could be an assistant method for pathologists in their practice and other laboratory tests like cytometry, Biomarkers and others.

Many 3D CNN models were designed to distinguish between benign and malignant nodules. Hussein et al. have used a standard 3D CNN model with an accuracy of 91.26%. In addition, Kang et al. designed a 3D multi‐view CNN.^69^ In a systematic review and meta‐analysis carried out on 26 studies, comprising 150,721 imaging data, Thong et al. (2023), demonstrated that AI‐based imaging had a pooled sensitivity of 94.6% and specificity of 93.6% for lung cancer screening.^70^ Rezayi et al. (2022), analyzed 63 studies in a systematic review to evaluate the use of AI techniques in neoplasm precision medicine. Seventeen articles out of 63 used Linear and nonlinear categories (random forest or decision trees) as the main AI approaches. In addition, rule‐based systems and DL models were used in 21 citations. Breast and lung cancers were predominantly selected in the 63 papers. The results of this study found that the highest values of predictors such as accuracy, sensitivity, specificity, precision, recall, and AUC were 0.99, 1.00, 0.96, 0.98, 0.99, and 0.9929, respectively. These findings suggest that AI techniques have a significant role in neoplasm precision medicine, particularly in personalized medicine^71^ (Figure 2).

#### 3.4.1 AI‐based CT scan

Many studies appreciated the value of AI assisting radiologists in detecting lung nodules and malignancy prediction on CT scans. Abadia et al. demonstrated similar sensitivity of an experienced radiologist and AI‐CNN model in detecting lung nodules on CT in patients with complex lung disease. Moreover, CNN helped to identify missed nodules.^72^ With AI assistance, radiologists achieved a higher sensitivity and AUC, but a bit lower specificity in detection. Furthermore, they achieved a higher sensitivity, specificity, and AUC in terms of malignancy prediction.^73^ Another systematic review and meta‐analysis confirmed the accuracy of CNN models in detecting pulmonary nodules on CT images with sensitivity, specificity, and AUC of 0.93, 0.95, 0.98, respectively. However, there were some variability among the studies.^74^ Zhang et al. developed a predictive model (PKU‐ML model) that can predict malignancy in multiple solid nodule patients (AUC = 0.838 in the test set) better than the previous models. This model also achieved good performance in single solid nodule patients (AUC = 0.786).^75^ Chen et al. conducted a retrospective, controlled, nonrandomized, study, which included 104 patients with suspected NSCLC confirmed by pathology. AI reading system of pulmonary nodules was used. The software (deep‐wise healthcare V.1.3.0.1) was provided based on a deep learning model. Two radiologists evaluated nodules detected by this system. The results were compared with the original CT reports. Statistical analysis showed a huge increase of sensitivity with the use of AI reading system of pulmonary nodules, (72.94% without AI assistance, 94.12% with AI assistance), but clearly higher false positive rate (7.93% with radiologists, 22.22% with AI), This suggests that AI film reading system based on deep learning could be an important assistant tool to screen for NSCLC.^76^

#### 3.4.2 Digital tomosynthesis

Digital tomosynthesis is a modality of CT scan but with reduced radiation and cost. This technique permits reconstruction of any plane in the patient overcoming the conventional tomography which is limited to a single plane reconstruction.^77^ Chauvie et al. compared the efficacy of different approaches in improving the PPV of chest digital tomosynthesis (DTS) for lung cancer detection. These approaches included binary visual analysis, lung‐RADS classification, logistic regression, random forest, and neural network. The study found that neural network (NNET) was the most effective technique in enhancing (PPV), with a PPV of 0.95 and a high sensitivity of 0.90.^78^

#### 3.4.3 AI‐Based radiography

Chest radiographs are an inexpensive screening method for detecting lung cancer. However, a radiologist can sometimes miss a nodule. Nam et al. combined computer‐aided detection (CAD) with a picture archiving and communication system (PACS) in their prospective trial. A total of 10,476 participants were randomized to an AI group (intervention group) and non‐AI group (control). All participants were exposed to a chest radiograph and were followed up later they demonstrated a high detection rate of actionable nodules in the AI group compared to control (0.59% VS 0.25%), respectively (*p*‐value = 0.008). Moreover, the detection rate of malignant lung nodules in the CAD‐based group was significantly improved (0.15%) compared with non‐AI group (0.00%), (*p*‐value = 0.008).^79^ A clinical trial designed to test DL‐based algorithm (Lunit INSIGHT CXR version 2.0, Lunit Inc.) in assisting radiologists detect Lung‐RADS category 4 nodules on chest radiographs showed improved sensitivity, although detection rate difference was only 0.24.^80^

#### 3.4.4 AI‐based pathology and cytology

The accurate diagnosis of lung cancer is by histology combined with molecular data. However, limited available diagnostic material, lack of pathologists globally, subjectivity of diagnosis, and intra‐ and inter‐observer variability are concerning limitations. Hence, digital pathology could be an assistant method for pathologists in their practice. In their systematic review, Davri et al. mentioned the existing AI‐based models that use pathologic and cytologic images. Most of these models were designed to distinguish between lung adenocarcinoma, lung SCC and small cell lung cancer with several studies centering on figuring out the predominant architectural pattern of adenocarcinoma, prognosis prediction, mutations and programmed cell death ligand 1 (PD‐L1) expression.^81^

#### 3.4.5 AI‐based routine laboratory data

Laboratory data is an easy, noninvasive method and has the potential to be used in the early detection of lung cancer. One retrospective observational cohort study was performed by Wei et al. Their study aimed to develop an AI model for early differentiation between malignant pleural effusion (MPE) and benign pleural effusion using clinical data which included age, sex, and laboratory parameters (hematologic and pleural fluid biomarkers). Five machine learning models were extracted from these data. The XGBoost model demonstrated the highest AUC results, exceeding carcinoembryonic antigen in the diagnosis of lung cancer‐induced MPE.^82^

#### 3.4.6 AI‐based breath test

Based on the fact that oxidative stress increases the production of reactive oxygen species and polyunsaturated fatty acids peroxidation, specific volatile organic compounds are produced. These compounds could be measured in the breath. Huang et al. conducted a prospective study to detect lung cancer using a breath test. In their trial, they analyzed alveolar air samples with carbon nanotube sensor arrays. They extracted a model using linear discriminant analysis (LDA) and nonlinear support vector machine (SVM) learning techniques. The reference for diagnostic accuracy was pathological reports. In the external validation, the area under the receiver operating characteristic curve was 0.91 using LDA and 0.90 using SVM.^83^

#### 3.4.7 AI‐bases flow cytometry

Lemieux et al. (2023) developed an improvised sputum‐based test (CyPath Lung), which is a combination of flow cytometry and machine learning. This test is intended for application after lung cancer screening to classify patients with cancer. The test demonstrated high accuracy, achieving an area under the ROC curve (AUC), sensitivity, specificity, negative and positive predictive values of 0.89, 82%, 88%, 96%, and 61%, respectively.^84^

The armamentarium of imaging, clinical, and laboratory data with AI models offered revolutionary outcomes in lung cancer early detection. Although there are some limitations, AI techniques in clinical practice, researchers expect it to play a huge role in the future of medical diagnosis.

## 4 CONCLUSION

In this review, we did a comprehensive study of the latest screening methods for NSCLC to assess which of these methods is most efficient in achieving early diagnosis. Some new procedures have shown a high sensitivity and specificity for early‐stage NSCLC detection. EB‐OCT, for instance, has a varied sensitivity and specificity—an average of 94.3% and 89.9 for each. On the other hand, detecting biomarkers via liquid biopsy carries an average sensitivity of 91.4% for RNA molecules detection, and 97% for combined methylated DNA panels. However, it is well known that the previous methods could be highly expensive as screening approaches, especially since the screening population is not specified. Moreover, CTCs detection did not prove to have a significant role as a screening method due to the rarity of CTCs in the bloodstream thus the need for more blood samples and for enrichment techniques. Hence, LDCT is still the golden standard and the only recommended screening procedure for its high sensitivity and specificity and proven cost‐effectiveness. Nevertheless, the notable false positive results acquired during the LDCT examination caused a presumed concern which drives researchers to investigate better screening procedures and approaches, particularly with the rise of the AI era or by combining two methods in a well‐studied screening program like LDCT and liquid biopsy. The cost‐effectiveness of lung cancer screening will mostly depend on the accurate selection of the target population and the optimal application of these new techniques. Thus, we suggest conducting more clinical studies on larger populations with a clear demographical target and adopting approaches for combining one of these new methods with LDCT to decrease false‐positive cases in early detection.

## AUTHOR CONTRIBUTIONS

**Nour Kenaan:** Project administration (lead); resources (equal); writing – original draft (equal). **George Hanna:** Resources (equal); writing – original draft (equal). **Moustafa Sardini:** Resources (equal); writing – original draft (equal). **Mhd Omar Iyoun:** Resources (equal); writing – original draft (equal). **Khedr Layka:** Resources (supporting); software (supporting); writing – original draft (supporting).

## FUNDING INFORMATION

None.

## CONFLICT OF INTEREST STATEMENT

The authors declare no conflict of interest.

## ETHICS STATEMENT

Not applicable.

## Data Availability

Data sharing is not applicable to this article as no new data were created or analyzed in this study.

## References

[1] Healthcare . Free Full‐Text | Systematic Review of Lung Cancer Screening: Advancements and Strategies for Implementation [Internet]. 2023 Available from: https://www.mdpi.com/2227‐9032/11/14/2085 10.3390/healthcare11142085 PMC10379173 37510525

[2] Accuracy of minimal residual disease detection by circulating tumor DNA profiling in lung cancer: a meta‐analysis. BMC Med. 2023;21:180. doi:10.1186/s12916-023-02849-z 37173789 PMC10176776

[3] Hao J , Shen Z . A systematic review and meta‐analysis of the diagnostic value of circulating microRNA‐17‐5p in patients with non‐small cell lung cancer. Medicine (Baltimore). 2023;102(8):e33070. 36827064 10.1097/MD.0000000000033070 PMC11309709

[4] Mazzone PJ , Silvestri GA , Souter LH , et al. Screening for lung cancer. Chest. 2021;160(5):e427‐e494. 34270968 10.1016/j.chest.2021.06.063 PMC8727886

[5] El‐Aal AEA , Elshafei A , Ismail MY , El‐Shafey MM . Identification of miR‐106b‐5p, miR‐601, and miR‐760 expression and their clinical values in non‐small cell lung cancer (NSCLC) Patients' serum. Pathol Res Pract. 2023;1(248):154663. 10.1016/j.prp.2023.154663 37429174

[6] Panunzio A , Sartori P . Lung Cancer and Radiological Imaging. Curr Radiopharm. 2020;13(3):238‐242. 32445458 10.2174/1874471013666200523161849 PMC8206195

[7] Sim AJ , Kaza E , Singer L , Rosenberg SA . A review of the role of MRI in diagnosis and treatment of early stage lung cancer. Clin Transl Radiat Oncol. 2020;1(24):16‐22. 10.1016/j.ctro.2020.06.002 PMC7306507 32596518

[8] Added value of respiratory gating in positron emission tomography for the clinical Management of Lung Cancer Patients ScienceDirect. 2024;52:745‐758. Available from: https://www.sciencedirect.com/science/article/pii/S000129982200037X?via%3Dihub 10.1053/j.semnuclmed.2022.04.006 35643531

[9] Owens C , Hindocha S , Lee R , Millard T , Sharma B . The lung cancers: staging and response, CT, 18F‐FDG PET/CT, MRI, DWI: review and new perspectives. Br J Radiol. 2023;96(1148):20220339. 37097296 10.1259/bjr.20220339 PMC10392646

[10] Behr CM , Oude Wolcherink MJ , IJzerman MJ , Vliegenthart R , Koffijberg H . Population‐based screening using low‐dose chest computed tomography: a systematic review of health economic evaluations. PharmacoEconomics. 2023 Apr;41(4):395‐411. 36670332 10.1007/s40273-022-01238-3 PMC10020316

[11] Prognostic value of consolidation‐to‐tumor ratio on computed tomography in NSCLC: a meta‐analysis. World Journal of Surgical Oncology. 2023;21:190. doi:10.1186/s12957-023-03081-y 37349739 PMC10286506

[12] Kan CFK , Unis GD , Li LZ , et al. Circulating biomarkers for early stage non‐small cell lung carcinoma detection: supplementation to low‐dose computed tomography. Front Oncol. 2021;11. doi:10.3389/fonc.2021.555331 PMC8099172 33968710

[13] Agrawal S , Goel AD , Gupta N , Lohiya A . Role of low dose computed tomography on lung cancer detection and mortality—an updated systematic review and meta‐analysis. Monaldi arch chest dis. Arch Monaldi Mal Torace. 2022;93(1). 10.4081/monaldi.2022.2284 35727220

[14] Dickson JL , Horst C , Nair A , Tisi S , Prendecki R , Janes SM . Hesitancy around low‐dose CT screening for lung cancer. Ann Oncol off J Eur Soc Med Oncol. 2022;33(1):34‐41. 10.1016/j.annonc.2021.09.008 34555501

[15] Gierada DS , Black WC , Chiles C , Pinsky PF , Yankelevitz DF . Low‐dose CT screening for lung cancer: evidence from 2 decades of study. Radiol Imaging Cancer. 2020;2(2):e190058. 32300760 10.1148/rycan.2020190058 PMC7135238

[16] Nooreldeen R , Bach H . Current and future development in lung cancer diagnosis. Int J Mol Sci. 2021;22(16):8661. 34445366 10.3390/ijms22168661 PMC8395394

[17] Toumazis I , de Nijs K , Cao P , et al. Cost‐effectiveness evaluation of the 2021 US preventive services task force recommendation for lung cancer screening. JAMA Oncol. 2021;7(12):1833‐1842. 34673885 10.1001/jamaoncol.2021.4942 PMC8532037

[18] Kalinke L , Thakrar R , Janes SM . The promises and challenges of early non‐small cell lung cancer detection: patient perceptions, low‐dose CT screening, bronchoscopy and biomarkers. Mol Oncol. 2021;15(10):2544‐2564. 33252175 10.1002/1878-0261.12864 PMC8486568

[19] Yang P . PS01.02 National Lung Cancer Screening Program in Taiwan: the TALENT study. J Thorac Oncol. 2021;16(3):S58.

[20] Chang GC , Chiu CH , Yu CJ , et al. Low‐dose CT screening among never‐smokers with or without a family history of lung cancer in Taiwan: a prospective cohort study. Lancet Respir Med. 2024;12(2):141‐152. 38042167 10.1016/S2213-2600(23)00338-7

[21] In vivo polarisation sensitive optical coherence tomography for fibrosis assessment in interstitial lung disease: a prospective, exploratory, observational study | BMJ Open Respir Res. 2023;10. Available from: https://bmjopenrespres.bmj.com/content/10/1/e001628 10.1136/bmjresp-2023-001628 PMC10414088 37553184

[22] Frontiers . EB‐OCT: a potential strategy on early diagnosis and treatment for lung cancer [Internet]. 2023. doi:10.3389/fonc.2023.1156218/full PMC10168178 37182131

[23] Validation of human small airway measurements using endobronchial optical coherence tomography respiratory medicine. 2023 Available from: https://www.resmedjournal.com/article/S0954‐6111(15)30054‐8/fulltext. 10.1016/j.rmed.2015.09.006 26427628

[24] Optical coherence tomography for identification of malignant pulmonary nodules based on random forest machine learning algorithm. PLOS ONE [Internet]. 2023;16. doi:10.1371/journal.pone.0260600 PMC8719667 34971557

[25] Hariri LP , Mino‐Kenudson M , Applegate MB , et al. Toward the guidance of Transbronchial biopsy. Chest. 2013;144(4):1261‐1268. 23828441 10.1378/chest.13-0534 PMC3787917

[26] Lam S , Standish B , Baldwin C , et al. In vivo optical coherence tomography imaging of Preinvasive bronchial lesions. Clin Cancer Res. 2008;14(7):2006‐2011. 18381938 10.1158/1078-0432.CCR-07-4418 PMC2849640

[27] Zhu Q , Yu H , Liang Z , et al. Novel image features of optical coherence tomography for pathological classification of lung cancer: results from a prospective clinical trial. Front Oncol. 2022;21(12):870556. 10.3389/fonc.2022.870556 PMC9634220 36338729

[28] Bronchoscopic needle‐based confocal laser endomicroscopy (nCLE) as a real‐time detection tool for peripheral lung cancer ‐ PubMed [Internet]. 2024 Available from: https://pubmed.ncbi.nlm.nih.gov/34172559/ 10.1136/thoraxjnl-2021-216885 PMC8938671 34172559

[29] Sawada T , Takizawa H , Aoyama M , et al. Diagnosis of visceral pleural invasion using confocal laser endomicroscopy during lung cancer surgery. J Thorac Dis. 2021;13(8):4742‐4752. 34527315 10.21037/jtd-21-137 PMC8411182

[30] Manley CJ , Kramer T , Kumar R , et al. Robotic bronchoscopic needle‐based confocal laser endomicroscopy to diagnose peripheral lung nodules. Respirol Carlton Vic. 2023;28(5):475‐483. 10.1111/resp.14438 PMC11590500 36535801

[31] Rapid non‐destructive volumetric tumor yield assessment in fresh lung core needle biopsies using polarization sensitive optical coherence tomography ‐ PMC [Internet]. 2024 Available from: https://www.ncbi.nlm.nih.gov/pmc/articles/PMC8515979/ 10.1364/BOE.433346 PMC8515979 34692203

[32] Ghigna MR , Crutu A , Florea V , et al. Endobronchial ultrasound‐guided fine‐needle aspiration for pulmonary carcinomas genotyping: experience with 398 cases including rapid EGFR/KRAS analysis in 43 cases. J Thorac Dis. 2018;10(7):4653‐4658. 30174918 10.21037/jtd.2018.06.157 PMC6105967

[33] Li W , Liu JB , Hou LK , et al. Liquid biopsy in lung cancer: significance in diagnostics, prediction, and treatment monitoring. Mol Cancer. 2022;21(1):25. 35057806 10.1186/s12943-022-01505-z PMC8772097

[34] Chang L , Li J , Zhang R . Liquid biopsy for early diagnosis of non‐small cell lung carcinoma: recent research and detection technologies. Biochim Biophys Acta BBA ‐ Rev Cancer. 2022;1877(3):188729. 10.1016/j.bbcan.2022.188729 35436521

[35] Abbasian MH , Ardekani AM , Sobhani N , Roudi R . The role of genomics and proteomics in lung cancer early detection and treatment. Cancer. 2022;14(20):5144. 10.3390/cancers14205144 PMC9600051 36291929

[36] Zhang C , Kim RY , McGrath CM , et al. The performance of an extended next generation sequencing panel using Endobronchial ultrasound‐guided fine needle aspiration samples in non‐squamous non‐small cell lung cancer: a pragmatic study. Clin Lung Cancer. 2023;24(2):e105‐e112. 36599742 10.1016/j.cllc.2022.11.010 PMC10664188

[37] Gu X , He J , Ji G . Combined use of circulating tumor cells and salivary mRNA to detect non–small‐cell lung cancer. Medicine (Baltimore). 2020;99(8):e19097. 32080083 10.1097/MD.0000000000019097 PMC7034687

[38] Gilad S , Lithwick‐Yanai G , Barshack I , et al. Classification of the four main types of lung cancer using a microRNA‐based diagnostic assay. J Mol Diagn. 2024;14:510‐517. https://www.jmdjournal.org/article/S1525‐1578(12)00125‐0/fulltext 10.1016/j.jmoldx.2012.03.004 22749746

[39] Huang L , Rong Y , Tang X , Yi K , Wu J , Wang F . Circular RNAs are promising biomarkers in liquid biopsy for the diagnosis of non‐small cell lung cancer. Front Mol Biosci. 2023;8. doi:10.3389/fmolb.2021.625722 PMC8201604 34136531

[40] Ding C , Xi G , Wang G , et al. Exosomal circ‐MEMO1 promotes the progression and aerobic glycolysis of non‐small cell lung cancer through targeting MiR‐101‐3p/KRAS axis. Front Genet. 2020;11:962. doi:10.3389/fgene.2020.00962 33005174 PMC7483554

[41] Pedraz‐Valdunciel C , Giannoukakos S , Potie N , et al. Digital multiplexed analysis of circular RNAs in FFPE and fresh non‐small cell lung cancer specimens. Mol Oncol. 2022;16(12):2367‐2383. 35060299 10.1002/1878-0261.13182 PMC9208080

[42] Schildhaus HU . Immunohistochemistry‐based predictive biomarkers for lung cancer. Pathologica. 2020;41(1):21‐31. 10.1007/s00292-020-00750-7 31989233

[43] Šutić M , Vukić A , Baranašić J , et al. Diagnostic, predictive, and prognostic biomarkers in non‐small cell lung cancer (NSCLC) management. J Pers Med. 2021;11(11):1102. 34834454 10.3390/jpm11111102 PMC8624402

[44] Ericson Lindquist K , Gudinaviciene I , Mylona N , et al. Real‐world diagnostic accuracy and use of Immunohistochemical markers in lung cancer diagnostics. Biomol Ther. 2021;11(11):1721. 10.3390/biom11111721 PMC8615395 34827719

[45] Dama E , Colangelo T , Fina E , et al. Biomarkers and lung cancer early detection: state of the art. Cancer. 2021;13(15):3919. 10.3390/cancers13153919 PMC8345487 34359818

[46] Katz RL , Zaidi TM , Pujara D , et al. Identification of circulating tumor cells using 4‐color fluorescence in situ hybridization: validation of a noninvasive aid for ruling out lung cancer in patients with low‐dose computed tomography‐detected lung nodules. Cancer Cytopathol. 2020;128(8):553‐562. 32320527 10.1002/cncy.22278

[47] Jin F , Zhu L , Shao J , et al. Circulating tumour cells in patients with lung cancer universally indicate poor prognosis. Eur Respir Rev. 2022;31(166):220151. 36517047 10.1183/16000617.0151-2022 PMC9879327

[48] Manjunath Y , Upparahalli SV , Suvilesh KN , et al. Circulating tumor cell clusters are a potential biomarker for detection of non‐small cell lung cancer. Lung Cancer Amst Neth. 2019;134:147‐150. 10.1016/j.lungcan.2019.06.016 31319973

[49] Borg M , Wen SWC , Andersen RF , Timm S , Hansen TF , Hilberg O . Methylated circulating tumor DNA in blood as a tool for diagnosing lung cancer: a systematic review and meta‐analysis. Cancer. 2023;15(15):3959. doi:10.3390/cancers15153959 PMC10417522 37568774

[50] Farooq M , Herman JG . Noninvasive diagnostics for early detection of lung cancer: challenges and potential with a focus on changes in DNA methylation. Cancer Epidemiol Biomarkers Prev. 2020;29(12):2416‐2422. doi:10.1158/1055-9965.EPI-20-0704 33148791 PMC11559093

[51] Wen SWC , Wen J , Hansen TF , Jakobsen A , Hilberg O . Cell free methylated tumor DNA in bronchial lavage as an additional tool for diagnosing lung vancer – a systematic review. Cancer. 2022;14(9):2254. doi:10.3390/cancers14092254 PMC9099950 35565384

[52] Banga I , Paul A , Sardesai AU , Muthukumar S , Prasad S . ZEUS (ZIF‐based electrochemical ultrasensitive screening) device for isopentane analytics with focus on lung cancer diagnosis. RSC Adv. 2021;11(33):20519‐20528. doi:10.1039/d1ra03093k 35479925 PMC9033977

[53] Rai D , Pattnaik B , Bangaru S , et al. MicroRNAs in exhaled breath condensate: a pilot study of biomarker detection for lung cancer. Cancer Treat Res Commun. 2023;35:100689. 36773435 10.1016/j.ctarc.2023.100689

[54] Liang R , Li X , Li W , Zhu X , Li C . DNA methylation in lung cancer patients: opening a “window of life” under precision medicine. Biomed Pharmacother. 2021;1(144):112202. 10.1016/j.biopha.2021.112202 34654591

[55] Thierry AR . Circulating DNA fragmentomics and cancer screening. Cell Genomics. 2023;3(1):100242. 36777187 10.1016/j.xgen.2022.100242 PMC9903826

[56] Che H , Jiang P , Choy LYL , et al. Genomic origin, fragmentomics, and transcriptional properties of long cell‐free DNA molecules in human plasma. Genome Res. 2024;34(2):189‐200. 38408788 10.1101/gr.278556.123 PMC10984381

[57] Zhang Z , Pi X , Gao C , et al. Integrated fragmentomic profile and 5‐Hydroxymethylcytosine of capture‐based low‐pass sequencing data enables pan‐cancer detection via cfDNA. Transl Oncol. 2023;18(34):101694. 10.1016/j.tranon.2023.101694 PMC10209323 37209526

[58] Martínez‐Reyes I , Chandel NS . Cancer metabolism: looking forward. Nat Rev Cancer. 2021;21(10):669‐680. 34272515 10.1038/s41568-021-00378-6

[59] Wang Y , Patti GJ . The Warburg effect: a signature of mitochondrial overload. Trends Cell Biol. 2023;33(12):1014‐1020. 37117116 10.1016/j.tcb.2023.03.013 PMC10600323

[60] DeBerardinis RJ , Chandel NS . We need to talk about the Warburg effect. Nat Metab. 2020;2(2):127‐129. 32694689 10.1038/s42255-020-0172-2

[61] Mariën H , Derveaux E , Vanhove K , Adriaensens P , Thomeer M , Mesotten L . Changes in metabolism as a diagnostic tool for lung cancer: systematic review. Meta. 2022;12(6):545. 10.3390/metabo12060545 PMC9229104 35736478

[62] Haince JF , Joubert P , Bach H , Ahmed Bux R , Tappia PS , Ramjiawan B . Metabolomic fingerprinting for the detection of early‐stage lung cancer: from the genome to the metabolome. Int J Mol Sci. 2022;23(3):1215. 35163138 10.3390/ijms23031215 PMC8835988

[63] Lee KB , Ang L , Yau WP , Seow WJ . Association between metabolites and the risk of lung cancer: a systematic literature review and meta‐analysis of observational studies. Meta. 2020;10(9):362. 10.3390/metabo10090362 PMC7570231 32899527

[64] Zhu M , Zeng Q , Fan T , et al. Clinical significance and Immunometabolism landscapes of a novel recurrence‐associated lipid metabolism signature in early‐stage lung adenocarcinoma: a comprehensive analysis. Front Immunol. 2022;13:783495. 35222371 10.3389/fimmu.2022.783495 PMC8867215

[65] P W, Q H, S M, T M, Z L, M H . Identification of lung cancer breath biomarkers based on perioperative breathomics testing: a prospective observational study. EClinicalMedicine. 2022;47. Available from: https://pubmed.ncbi.nlm.nih.gov/35480076/ 10.1016/j.eclinm.2022.101384 PMC9035731 35480076

[66] Lung Nodule Detection from Feature Engineering to Deep Learning in Thoracic CT Images: a Comprehensive Review ‐ PubMed [Internet]. 2023 Available from: https://pubmed.ncbi.nlm.nih.gov/31997045/ 10.1007/s10278-020-00320-6 PMC7256172 31997045

[67] Cellina M , Cè M , Irmici G , et al. Artificial intelligence in lung cancer imaging: unfolding the future. Diagn Basel Switz. 2022;12(11):2644. 10.3390/diagnostics12112644 PMC9689810 36359485

[68] Chassagnon G , De Margerie‐Mellon C , Vakalopoulou M , et al. Artificial intelligence in lung cancer: current applications and perspectives. Jpn J Radiol. 2023;41(3):235‐244. 36350524 10.1007/s11604-022-01359-x PMC9643917

[69] Li C , Wang H , Jiang Y , et al. Advances in lung cancer screening and early detection. Cancer Biol Med. 2022;19(5):591‐608. 35535966 10.20892/j.issn.2095-3941.2021.0690 PMC9196057

[70] Thong LT , Chou HS , Chew HSJ , Lau Y . Diagnostic test accuracy of artificial intelligence‐based imaging for lung cancer screening: a systematic review and meta‐analysis. Lung Cancer Amst Neth. 2023;176:4‐13. 10.1016/j.lungcan.2022.12.002 36566582

[71] Effectiveness of Artificial Intelligence for Personalized Medicine in Neoplasms: A Systematic Review ‐ PubMed [Internet]. 2023 Available from: https://pubmed.ncbi.nlm.nih.gov/35434134/ 10.1155/2022/7842566 PMC9010213 35434134

[72] Abadia AF , Yacoub B , Stringer N , et al. Diagnostic accuracy and performance of artificial intelligence in detecting lung nodules in patients with complex lung disease: a noninferiority study. J Thorac Imaging. 2022;37(3):154‐161. 34387227 10.1097/RTI.0000000000000613

[73] Ewals LJS , van der Wulp K , van den Borne BEEM , et al. The effects of artificial intelligence assistance on the Radiologists' assessment of lung nodules on CT scans: a systematic review. J Clin Med. 2023;12(10):3536. 37240643 10.3390/jcm12103536 PMC10219568

[74] Zhang X , Liu B , Liu K , Wang L . The diagnosis performance of convolutional neural network in the detection of pulmonary nodules: a systematic review and meta‐analysis. Acta Radiol Stockh Swed. 2023;64(12):2987‐2998. 10.1177/02841851231201514 37743663

[75] Zhang K , Wei ZH , Wang X , Chen KZ . The diagnostic value of machine‐learning‐based model for predicting the malignancy of solid nodules in multiple pulmonary nodules. Zhonghua Wai Ke Za Zhi. 2022;60(6):573‐579. 35658345 10.3760/cma.j.cn112139-20211101-00511

[76] Chen Y , Tian X , Fan K , Zheng Y , Tian N , Fan K . The value of artificial intelligence film Reading system based on deep learning in the diagnosis of non‐small‐cell lung cancer and the significance of efficacy monitoring: a retrospective, clinical, nonrandomized. Controlled Study Comput Math Methods Med. 2022;2022:2864170. 35360550 10.1155/2022/2864170 PMC8964156

[77] Ferrari A , Bertolaccini L , Solli P , Di Salvia PO , Scaradozzi D . Digital chest tomosynthesis: the 2017 updated review of an emerging application. Ann Transl Med. 2018;6(5):91. 29666814 10.21037/atm.2017.08.18 PMC5890049

[78] Chauvie S , De Maggi A , Baralis I , et al. Artificial intelligence and radiomics enhance the positive predictive value of digital chest tomosynthesis for lung cancer detection within SOS clinical trial. Eur Radiol. 2020;30(7):4134‐4140. 32166491 10.1007/s00330-020-06783-z

[79] Nam JG , Hwang EJ , Kim J , et al. AI improves nodule detection on chest radiographs in a health screening population: a randomized controlled trial. Radiology. 2023;307(2):e221894. 36749213 10.1148/radiol.221894

[80] Nam JG , Kim HJ , Lee EH , et al. Value of a deep learning‐based algorithm for detecting lung‐RADS category 4 nodules on chest radiographs in a health checkup population: estimation of the sample size for a randomized controlled trial. Eur Radiol. 2022;32(1):213‐222. 34264351 10.1007/s00330-021-08162-8

[81] Davri A , Birbas E , Kanavos T , et al. Deep learning for lung cancer diagnosis, prognosis and prediction using histological and cytological images: a systematic review. Cancer. 2023;15(15):3981. 10.3390/cancers15153981 PMC10417369 37568797

[82] Wei TT , Zhang JF , Cheng Z , Jiang L , Li JY , Zhou L . Development and validation of a machine learning model for differential diagnosis of malignant pleural effusion using routine laboratory data. Ther Adv Respir Dis. 2023;17:17534666231208632. 37941347 10.1177/17534666231208632 PMC10637149

[83] Huang CH , Zeng C , Wang YC , et al. A study of diagnostic accuracy using a chemical sensor Array and a machine learning technique to detect lung cancer. Sensors. 2018;18(9):2845. 30154385 10.3390/s18092845 PMC6164114

[84] Detection of early‐stage lung cancer in sputum using automated flow cytometry and machine learning ‐ PubMed [Internet]. 2023 Available from: https://pubmed.ncbi.nlm.nih.gov/36681813/ 10.1186/s12931-023-02327-3 PMC9862555 36681813

